# Dental anxiety and pain related to ART

**DOI:** 10.1590/S1678-77572009000700015

**Published:** 2009

**Authors:** Soraya Coelho LEAL, Danielle Matos de Menezes ABREU, Jo E. FRENCKEN

**Affiliations:** 1 MSc, PhD, Associate Professor, Department of Dentistry, School of Health Science, University of Brasília, Brasília, DF, Brazil.; 2 MSc, PhD student, Department of Dentistry, School of Health Science, University of Brasília, Brasília, DF, Brazil.; 3 DDS, MSc, PhD, Associate Professor, Department of Global Oral Health, Radboud University Nijmegen Medical Centre, College of Dental Sciences, Nijmegen, the Netherlands.

**Keywords:** Atraumatic Restorative Treatment (ART) Dental anxiety, Dental pain, Discomfort, Dental fear

## Abstract

Atraumatic Restorative Treatment (ART) is considered to be well accepted, both by children and by adult patients. The objective of this review is to present and discuss the evidence regarding the acceptability of ART, from the patient's perspective. Aspects related to dental anxiety/fear and pain/discomfort have been highlighted, to facilitate better understanding and use of the information available in the literature. Conclusions: The ART approach has been shown to cause less discomfort than other conventional approaches and is, therefore, considered a very promising “atraumatic” management approach for cavitated carious lesions in children, anxious adults and possibly, for dental-phobic patients.

## INTRODUCTION

The Atraumatic Restorative Treatment (ART) is a minimum intervention approach for managing carious lesions. Only hand instruments are used for cavity preparation and cleaning followed by restoration of the cavity and sealing pits and fissures with an adhesive material such as glass ionomer cement^7^.

The “atraumatic” component of the technique can be understood from different perspectives, such as those of tooth tissue preservation and patients' comfort. Undoubtedly, using only hand instruments to open and clean the cavity preserves more sound dental structure than does the traditional approach that recommends the use of the drill^24^. In this respect, the ART approach is definitely less traumatic to the tooth than the conventional method. It also has the capacity to be more comfortable for patients, as the noise and vibration related to the bur are absent. This “atraumatic” effect is further enhanced by the fact that local anesthesia is rarely used in the ART approach^8^^,^^10^. This indicates that ART is a treatment that inflicts only a low level of trauma upon the patient. Finally, because the patients are more relaxed when ART is used in treating them, the technique may also reduce operator stress during interaction with the patient; and therefore, prove less traumatic to dentists than traditional methods^13^.

The objective of this review is to present and discuss evidence regarding the acceptability of ART from the patient perspective. Aspects related to dental anxiety/fear and pain/discomfort will be highlighted in order to engender better understanding and use of the information available in the literature.

## ART ACCEPTABILITY: LITERATURE EVIDENCE

In general, results retrieved from different clinical trials, conducted in different regions of the world, show that ART is well accepted both by children and by adults treated in accordance with this approach^5^^,^^18^^,^^22^. Specific methodological designs have been developed in order to demonstrate its effectiveness in terms of reducing patients' dental anxiety and causing less pain than the traditional approaches cause.

To investigate pain associated with both ART (using hand instruments) and a conventional approach (using high and low speed handpieces), in the removal of carious tissues, at the end of the restorative session a group of adolescents were asked whether any pain was felt during treatment. The authors concluded that ART was less painful than the conventional restoration technique^18^. This finding is in agreement with that of Schriks and van Amerongen^19^ (2003), who concluded that children treated according to the ART approach experienced less discomfort than those treated with rotary instruments. In both cases local anesthesia was not used. Nevertheless, in the latter study discomfort was not individually reported by the patient, but was assessed through physiological measurements (heart rate) and behavioral observations on specific moments during the treatment (entrance, start, deep excavation, matrix placement, restoration and at the end of treatment). Analysis of behavioral observations and physiological measurements showed only a moderate correlation, while behavioral scores demonstrated that children from the ART group were more relaxed throughout all the treatment procedures than were children treated with rotary instruments. The physiological measurements were able to detect significant differences between the groups during deep excavation only. However, the intercorrelation between different ways of assessing dental anxiety is usually low, which can be explained by the multidimensional fear construct. each measurement technique taps into a unique part of the process^1^.

Due to structural characteristics of dentin, it is expected that more pain will be experienced in relation to deep cavities. This association was demonstrated in a study that aimed to determine the level of sensitivity related to cavity size and lesion depth, experienced by adolescents during ART cavity preparation^5^. The report of pain and discomfort was, in general, low; more frequently experienced in large than in small cavities and in cavities with the floor close to the pulp. Tubules extending through the dentin, that are greater in density near the pulp than at the outer periphery, are the pathway for sensitive stimuli transmission^14^. This explains the association of cavity depth and reports of pain.

Little information is available regarding pain and discomfort related to the ART approach for both adults and young children. Pain assessment is not easily performed in children, as they have difficulties in expressing their emotions and feelings^27^. This problem was described by Menezes Abreu, et al.^12^ (2009). Pain experience in a group of young children (4 to 7 years old) after they had been treated according to the ART approach was compared with that of a group treated in accordance with a conventional approach using rotary instruments with local anesthesia and rubber dam. Children from the ART group reported less pain than those from the conventional one. The second finding was that 4 year-old children reported more pain than children aged 5 to 7 years old, independently of the treatment provided. The authors observed that the youngest children had experienced some difficulty in interpreting the pain rating scale used in the study.

In discussing dental anxiety in relation to ART, two contradicting studies have been published^13^^,^^22^. Mickenautsch, et al.^13^ (2007) concluded that patients (children and adults) treated with the ART approach were less-anxious than those treated by traditional methods using the drill and bur. In this study, patients' anxiety levels were assessed immediately after the restorative session had been completed. Two different interpretations of the results are possible: either the patients experienced less trauma using ART and were therefore less anxious or the patients treated by the ART approach were initially less anxious than those treated according to the traditional approach, and thus experienced less trauma. If dental anxiety in this study would also have been assessed prior to the treatment, the treatment effect could have been established.

In the second study, the authors were not able to demonstrate any difference in dental anxiety levels amongst children from 3 treatment groups (traditional, ART and ART in combination with a chemomechanical caries removal gel). As in the previously discussed study, the dental anxiety assessment was performed at the end of the treatment session. This method does not follow the common way of assessing dental anxiety, which should be carried out before the start of the dental visit and not after it has been completed. This factor might be the reason for the contradictory findings of the two studies.

On the basis of the information provided, it can be concluded that dental/fear and pain/discomfort related to different restoratives procedures require further investigation. Studies should include confounding factors; such as: age, gender, operator influence and cultural aspects^6^^,^^19^. Furthermore, methodological aspects should be given due attention, as both fear/anxiety and pain/discomfort levels may also be influenced by subjective aspects like emotional responses and social determinants^10^. Lastly, fear/anxiety and pain/discomfort assessment instruments should be used according to the instructions described in the original protocols.

## ANXIETY, FEAR, PAIN AND DISCOMFORT ASSOCIATED WITH DENTISTRY

Dental anxiety can be defined as a feeling of apprehension about dental treatment, not necessarily related to a specific stimulus^6^, while dental fear is a normal emotional reaction to one or more specific threatening stimuli in the dental situation^9^. Both terms are currently being used interchangeably in the dental literature when referring to negative feelings related to dental treatment. According to Panksepp^17^ (1982), the difference between fear and anxiety seems to reflect only the intensity.

A critical literature review estimates that 9% of the world population suffers from dental fear/anxiety, with a decrease in prevalence as age increases^9^. The etiology of dental anxiety is multifactorial, being strongly correlated to a history of dental pain in both adults and children^15^^,^^26^. A comparison of anxious and non-anxious children demonstrated that fear was more strongly associated with children's experience of pain and trauma than with objective dental pathology^23^.

Dental anxiety/fear may negatively impact on a person's life. According to Cohen, et al.^4^ (2000), physiological impacts include fright response and feelings of exhaustion after dental appointments, while behavioral impacts include dental avoidance. It is well established that anxious individuals frequently avoid dental treatment, either by failing to appear for their dental appointments or by delaying dental visits for long periods of time^11^.

**Figure 1:**
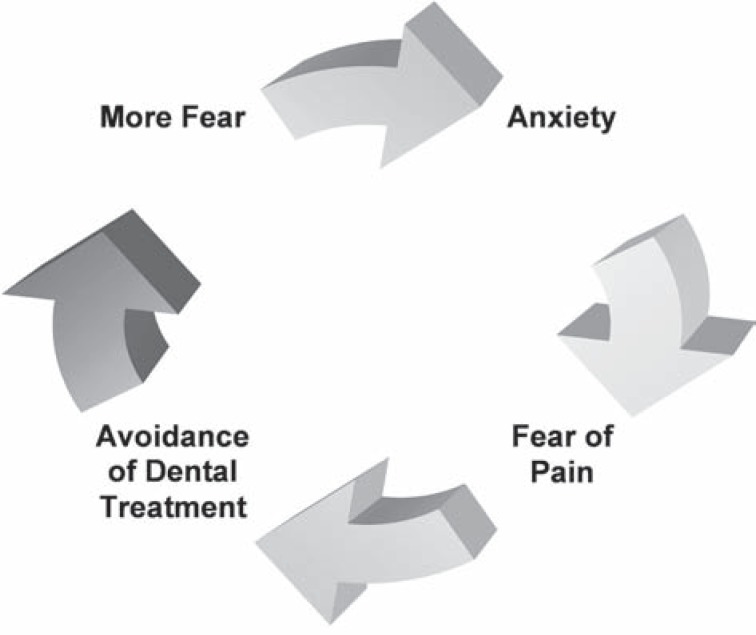
Vicious cycle of anxiety: modified from van Wijk and Hoogstraten^25^ (2009)

The interaction between anxiety and dental pain, as investigated by van Wijk and Hoogstraten^25^ (2005), suggests that people who respond fearfully to pain are at an increase risk of ending up in a vicious cycle of anxiety, as shown in Figure 1. If this cycle is not broken, a severe form of dental fear might develop. This can be defined, according to the Diagnostic and Statistical Manual of Mental Disorders (DSMIV), as a specific phobia - dental phobia. This phobia is characterized by marked and persistent anxiety in relation either to clear discernable situations (e.g.: drill, needle) or to the dental situation in general^3^.

Some interesting results related to the prevalence of dental fear and dental phobia in comparison to 10 other common fears and subtypes of specific phobia were reported in a recent investigation. The prevalence of dental fear was considered high (24.3%), but lower than that of fear of snakes, heights or physical injuries. Surprisingly, among the phobias, dental phobia was the most prevalent (3.7%)^16^. These findings should alert both researchers and dental practitioners to this very real issue with the objective of seeking ways to improve the condition.

Dental fear usually starts in childhood with a negative experience, commonly expressed as having had a painful event and/or being treated by a rough dentist^2^. Although it tends to decrease with an increase of age^9^, dental anxiety/fear can persist into middle and advanced adulthood^16^. It is essential, therefore, that dentists are capable to identify these patients, in order to plan the dental intervention that can reduce each individual's anxiety level.

## PERSPECTIVES: ART AS A TOOL FOR PATIENT MANAGEMENT

As previously discussed, dental fear is a potentially distressing condition: not only for the patient, but also for the dentist. The best strategy for dealing with this condition in children would be to employ appropriate pediatric management techniques that could assist the practitioner in identifying dental-anxious children as early as possible and to use dental interventions that cause the least possible psychological negativity.

The most common fear-inducing aspects of the dental treatment are the procedures related to the needle and the drill^20^^,^^21^. Individual vulnerability and perceptions of negative dentist behavior also play an important role in patients' dental anxiety development^2^.

In light of all these aspects, Atraumatic Restorative Treatment may become an important “tool” for managing carious dental lesions, both for young children and for anxious adults. The ART approach is based using only hand instruments to open the cavity and remove carious tissue^7^. This aspect may have a positive impact on patients' experience of discomfort, as the drill is not used. Because of that, the usual vibration and noise related to this equipment are not present and this facilitates better interaction between patient and dentist. In addition, because of removal of infected dentine only, local anesthesia is almost never required^13^. Thus, the ART technique is considered less traumatic, less painful and friendlier than the conventional restorative interventions. Further investigations, with well- designed research protocols are required in order to confirm these assumptions.

## CONCLUSIONS

Dental fear/anxiety and dental pain/discomfort are multifactorial phenomena that can negatively impact on an individual's life. Dentists should be able to identify, and be prepared to treat, fearful patients in a way that reduces their levels of dental anxiety. The ART approach has been shown to cause less discomfort than other conventional approaches and is, therefore, considered a very promising “atraumatic” management approach for cavitated carious lesions in children, anxious adults and possibly, for dental-phobic patients.
